# Effect of IDH3a on glucose uptake in lung adenocarcinoma: A pilot study based on [^18^F]FDG

**DOI:** 10.1002/cam4.2421

**Published:** 2019-07-29

**Authors:** Bulin Du, Tong Sun, Xuena Li, Yao Diao, Yaming Li

**Affiliations:** ^1^ Department of Nuclear Medicine The First Hospital of China Medical University Shenyang Liaoning China

**Keywords:** 2‐[^18^F]‐2‐deoxy‐D‐glucose, isocitrate dehydrogenase, lung adenocarcinoma, positron emission tomography

## Abstract

Subunit of isocitrate dehydrogenase 3 (IDH3a) as upstream of the hypoxia‐inducible factor was reported highly expressed in malignant tumors, playing an important role in glucose metabolism reprogramming. As one of rate‐limiting enzyme in the Krebs cycle, whether high expression of IDH3a affects glucose uptake in tumors has not been elucidated. This study was aimed to investigate the relationship between IDH3a expression and tumor glucose uptake. Sixty‐five patients who underwent 2‐[^18^F]‐2‐deoxy‐D‐glucose ([^18^F]‐FDG) positron emission tomography/computed tomography (PET/CT) imaging before surgery and pathologically diagnosed as lung adenocarcinoma were included. All patients were divided into high (n = 31) and low (n = 34) groups according IDH3a expression by immunohistochemistry. Comparatively higher [^18^F]‐FDG uptake was found in high IDH3a expression group. Glucose transporter 1 (GLUT1) level was demonstrated to correlate with IDH3a expression, but not for hexokinase 2 (HK2). Furthermore, A549 and H1299 cells experiment showed, the expression of p‐AKT and GLUT1 were significantly downregulated after IDH3a interference. The cellular uptake of [^18^F]‐FDG and lactate production were significantly reduced in treatment group. In summary, high expression of IDH3a in lung adenocarcinoma patients is associated with higher glucose uptake. IDH3a targets AKT‐GLUT1 pathway to affect glucose uptake and metabolites in lung adenocarcinoma.

## 1 INTRODUCTION

Reprogramming of glucose metabolism mainly refers to the generation of adenosine triphosphate (ATP) by glucose oxidative phosphorylation changing into aerobic glycolysis, which is a characteristic change of most malignant tumors. Conversely, abnormal glucose metabolism (such as mutations in key enzyme genes) can also lead to malignant tumors.^1^, ^2^ Isocitrate dehydrogenase (IDH) is the rate‐limiting enzyme in the Krebs cycle that catalyzes oxidative decarboxylation of isocitrate to α‐ketoglutaric acid. IDH3 is one of three subtypes of IDH, and its α subunit is a catalytic subunit having oxidative dehydrogenation activity. Recent studies have found that IDH3a mediates the aerobic glycolysis of tumor cells by upregulating hypoxia‐inducible factors (HIF) and shows that lung or breast cancer patients with high IDH3a expression have a poor prognosis.^3^ IDH3a has also been found highly expressed in aflatoxin‐induced hepatocellular carcinoma and glioblastoma.^4^, ^5^ However, another study found that IDH3a expression is downregulated in cancer‐associated fibroblasts, providing metabolites for the growth of tumors via aerobic glycolysis.^6^ In any case, the studies above confirmed that abnormal expression of IDH3a led to reduction of α‐ketoglutarate, and it was concluded that IDH3a played a key role in tumor aerobic glycolysis.

Positron emission tomography with 2‐deoxy‐2‐[fluorine‐18]fluoro‐ D‐glucose integrated with computed tomography (^18^F‐FDG PET/CT) is a powerful imaging tool for the detection of various cancers, providing valuable functional information based on the increased glucose uptake and glycolysis of cancer cells. The main mechanisms of [^18^F]‐FDG uptake is involved with increased expression of glucose transporters of cell membrane and rate‐limiting glycolytic enzymes, such as glucose transporters 1 (GLUT1) and hexokinase 2 (HK2).^7^, ^8^ AKT is known as "Warburg kinase," which promotes tumor cells metabolic reprogramming and increases cell invasiveness.^9^

The aim of this study was to investigate the relationship between IDH3a expression and glucose uptake of tumor, and to explore and elucidate the involvement of IDH3a in the AKT pathway affecting glucose uptake by detecting GLUT1 and HK2.

## 2 MATERIAL AND METHODS

### 2.1 Patients and specimens

Patients who underwent preoperative [^18^F]‐FDG PET/CT imaging in the Department of Nuclear Medicine from June 2014 to June 2016 were included. From these cases, 65 patients who were pathologically diagnosed with lung adenocarcinoma, and did not receive chemotherapy or radiotherapy before imaging were finally selected. Tissues for immunohistochemical staining are available, along with corresponding clinical data, including age, sex, tumor size, and TNM staging. All patients provided written informed consent to use the tumor specimen and clinical data for the research. All procedures involving human studies were approved by the Ethics Committee of the First Hospital of China Medical University.

### 2.2 PET/CT imaging

All patients underwent [^18^F]‐FDG PET/CT imaging using an integrated PET/CT device (Biograph mCT, Siemens). Subsequent to 6 hours of fasting, PET/CT scanning was started 60 minutes after intravenous injection of [^18^F]‐FDG with a maximum activity of 3.7 MBq/kg. Immediately after CT scanning, subsequent PET images were acquired, covering the identical axial field of view. Imaging was performed with the patients breathing normally during both parts of the procedure. PET/CT fused images were obtained after CT attenuation correction and datasets filtered Back‐Projection reconstruction. Semi‐quantitative indexes representing [^18^F]‐FDG uptake, including maximum standard uptake value (SUV_max_) and mean standard uptake value (SUV_mean_), were automatically calculated based on the maximum and average activities in the volume of interest as a fraction of the injected [^18^F]‐FDG dose per body weight. Metabolic tumor volume (MTV)is defined as the cubic centimeter volume of voxels with SUV >50% of SUV_max_, total lesion glycolysis (TLG) is defined as the product of MTV and the SUV_mean_ of voxels within the MTV.^10^ PET/CT images were reviewed by two experienced nuclear medicine physicians.

### 2.3 Immunohistochemistry

Resected surgical tissue specimens were fixed with 10% neutral buffered formalin for 24 hours, then embedded with paraffin. Tissue specimens were cut into 5 μm slices and placed onto glass microscope slides. After dehydrated with xylene and rehydrated with alcohol, the slices were subjected to antigen repair with sodium citrate buffer heated to boiling using a microwave oven. Then the slices were treated with an immunohistochemistry kit (MXB, Cat. # KIT9710) step by step. Fresh 0.3% hydrogen peroxide solution was used for 10 minutes to block the endogenous peroxidase. After serum block, the slices were incubated with anti‐IDH3a (1:300, Cat. #ab58641), anti‐GLUT1 (1:200, Cat. #ab40084), or anti‐HK2 (1:200, Cat. #ab104836) antibodies (all Abcam) overnight at 4°C, respectively. Then, they were incubated with HRP‐labeled anti‐mouse/rabbit secondary antibody for 10 minutes at 37°C. Mixed avidin–biotinylated enzyme was used for 10 minutes at room temperature to prevent avidin saturation. Reaction product was visualized using DAB horseradish peroxidase color development Kit (MXB, Cat. # DAB‐003). A confocal laser microscope system (Nikon, Eclipse 80i) was used to observe the stained tissue, the brown color indicates positive staining. All tumor slides were examined randomly by two independent pathologists. Staining intensity was classified as: 0: no staining, 1: moderate staining and 2: strong staining; and the staining percentage scored as: 1: 1%‐24%, 2: 25%‐49%, 3: 50%‐74%, and 4: 75%‐100%. Percentage and intensity scores were multiplied to give a final score. Tumors with a final score ≥4 were considered to be tumors with specific protein overexpression.

### 2.4 Cell culture and transfection

Human lung adenocarcinoma A549 cells (Cell Bank of CAS, Cat. # TCHu150) and H1299 (Cell Bank of CAS, Cat. # TCHu160) purchased from the Chinese Academy of Sciences were cultured in DMEM/F12 = 1:1 (HyClone, Cat. # SH30023.01B, GE Healthcare) or RPMI‐1640 (HyClone, Cat. # SH30027.01, GE Healthcare) supplemented with 10% fetal bovine serum (CLARK Bioscience, Cat. # FB15011) at 37℃ 5% CO_2_. 2 × 10^5^ A549 or H1299 cells were seeded and cultured in six‐well cell culture plates until they reached approximately 80%‐90% confluency. Lipofectamine 3000 (Invitrogen, Cat. # L3000001) was used for transient transfections according to the manufacturer's protocols. The small interference RNA of *IDH3a* was used (Viewsolid Biotech, Cat. # NM_005530). SiRNA sequences of *IDH3a* are as follows: S1: 5′‐GACAGUAACUUUAAUUCCAtt‐3′; S2: 5′‐GAUGUACCUUGAUACAGUAtt‐3′; S3: 5′‐GUAUCAAGCUCAUCACCGAtt‐3′. 5′‐GGTGACTGACAAATTGCtt‐3′ was used as non‐targeting control siRNA. Treated A549 or H1299 cells were collected 48 hours after transfection for corresponding test.

### 2.5 Quantitative real‐time PCR

Total RNA was isolated from cell lines using TRIzol reagent (Thermo Fisher, Cat. # 15596026). RNA (800 ng) was reverse‐transcribed using PrimeScrip RT Reagent Kit with gDNA Eraser (Takara, Cat. # RR047A) with both oligo dT and random hexamers. Quantitative real‐time PCR was performed using SYBR Premix Ex Tag II (Takara, Cat. # RR820A) in a Real‐Time PCR System (Roche, Lightcycler 480) with an annealing temperature at 60°C. The fold change of relative expression of target genes was calculated using the 2^−ΔΔCt^ method with the cycle threshold range from 16 to 25. After log‐transformation of data, relative mRNA expression between treated group and NC group was compared. The primers were synthesized by Takara (Takara biomedical technology, Cat. # SYD16035). Actin was served as a normalizing control. The sequences are as follows:

*IDH3ɑ* forward, 5′‐ATCGGAGGTCTCGGTGTG‐3′, IDH3ɑ reverse, 5′‐AGGAGGGCTGTGGGATTC‐3′; *GLUT1* forward, 5′‐CTGGCATCAACGCTGTCTTC‐3′, *GLUT1* reverse: 5′‐GCCTATGAGGTGCTGGGTC‐3′; *HK2* forward, 5′‐GAGCCACCACTCACCCTACT‐3′, *HK2* reverse, 5′‐ACCCAAAGCACACGGAAGTT‐3′; *β‐actin* forward, 5′‐GCAGAAGGAGATCACTGCCCT‐3′, *β‐actin* reverse, 5′‐GCTGATCCACATCTGCTGGAA‐3′. Measurements were performed in three independent experiments.

### 2.6 Western blot analysis

A549 or H1299 cells (5 × 10^6^) were harvested when they reached approximately 80%‐90% confluency, and lysed in western blot lysis buffer (Beyotime Biotech, Cat. # P0013) containing 1 mmol/L phenylmethanesulfonylfluoride (Beyotime Biotech, Cat. # ST506). Total proteins were extracted from the lysate and quantified using a bicinchoninic acid protein assay (Beyotime Biotech, Cat. # P0012). 10% or 6% polyacrylamide gels were used based on the molecular weight of proteins to be tested. 20 μg of protein was loaded per well. After fractionation, proteins were transferred to polyvinylidene fluoride membranes (EMD Millipore, Cat. # IPVH00010) using standard wet transfer method. After blocked with 5% non‐fat milk for 2 hours, membranes were incubated with primary antibodies in TBST overnight at 4°C. The following primary antibodies were used: anti‐IDH3a antibody (1:500; Cat. #yt5318), anti‐GLUT1 antibody (1:500; Cat. #yt1928), and anti‐HSP90 (1:500; Cat. #yt2257) (all Immunoway). HK2 antibody (1:1000; Cat. #ab209847), anti‐AKT antibody (1:1000; Cat. # ab32505), anti‐p‐AKT(T308) antibody (1:1000; Cat. # ab38449) and anti‐GADPH (1:10000; Cat. #ab181602) (all Abcam). After washing with TBST, membranes were incubated with secondary antibody (1:8000; Cat. #A23930) at 37°C for 2 hours. Immunoreactive bands were detected by enhanced chemiluminescence ECL (Thermo Fisher, Cat. # 32106), and scanned in the Gel Doc XR system (Bio‐Rad, Hercules). Images were analyzed with Image Lab software (version 3.0；Bio‐Rad). All experiments were performed in triplicate.

### 2.7 [^18^F] ‐FDG uptake

Cell uptake experiments were performed with [^18^F]‐FDG to assess glucose uptake with A549 or H1299 cells. Cells were cultured in six‐well cell culture plates until they reached approximately 80%‐90% confluency, then transfected with *IDH3a* siRNA. 48 hours after transfection, washed with PBS for three times, tumor cells were incubated in 2 mL DMEM containing [^18^F]‐FDG (148 kBq [4 μCi/mL]) for 1 hour at 37°C. Whole‐cell lysates were produced using 1 mL of trypsin‐EDTA, and the radioactivity was determined using a well γ‐counter (ZonKia). These readouts were normalized to corresponding protein amounts. All tests were performed in independent triplicate experiments.

### 2.8 Cell metabolism assay

To study changes in cellular metabolism, cellular adenosine triphosphate (ATP) and lactate levels in the medium were measured. Cells were seeded into six‐well plates for transfection until they reached approximately 80%‐90% confluency. Forty‐eight hours after transfection with *IDH3a* siRNA, the culture medium was collected for the measurement of the lactate concentration. The tumor cells were washed, centrifuged, and lysed. Lysates were collected for measurement of ATP levels. Relative cellular ATP content was measured using an ATP assay kit (Beyotime Biotech, Cat. # S0026) according to the manufacturer's instructions. Chemiluminescence was read using a multimode microplate reader (Thermo Fisher, Varioskan Flash). The lactate concentrations were measured using a Lactate assay kit (KeyGen, Cat. # KGT023) according to the manufacturer's instructions. Optical densities at 530 nm were read using a microplate reader (Thermo Fisher, Varioskan Flash). Measurements were performed in three independent experiments.

### 2.9 Cell proliferation assay

Cell proliferation was checked using a Cell Counting Kit‐8 (Dojindo Molecular Technologies, Cat. # CK0405). The cells were seeded at 5000 cells/well in 96‐well plates at 200 μL per well after 24 hours of transfection in three plates. Each well added 10 μL CCK‐8 after incubation for 24, 48, 72 hours, respectively, and incubated for another 4 hours. The optical density (OD) value at a wavelength of 450 nm was determined using an enzyme microplate reader (Thermo Fisher, Varioskan Flash). All experiments were performed in triplicate.

### 2.10 Bioinformatics analysis

To explore and further investigate the relationship between *IDH3a* expression and related indicators (GLUT1 and HK2), bioinformatics analysis with gene expression database including large samples was introduced. GEPIA (a web server for cancer and normal gene expression profiling and interactive analyses, http://gepia.cancer-pku.cn/) was used.^11^

### 2.11 Statistical analysis

Continuous variables were analyzed by the two‐tailed Student's *t* test. Box‐Whisker plots were used to evaluate differences of semi‐quantitative indexes in different protein expression groups. Median levels and interquartile ranges were illustrated by box plot, minimum and maximum values were shown as whiskers, and outliers were displayed as points. RNA expression, cellular glucose uptake and metabolites production were presented as mean ± standard deviation and shown in histograms with error bars. Dichotomous variables were analyzed by the χ^2^ test. A *P*‐value <.05 was considered to be significant. In addition to calculating the Phi coefficient using SPSS 20, other statistical analyses were performed using GraphPad Prism 5 statistical software.

## 3 RESULTS

### 3.1 Correlation between [^18^F]‐FDG uptake and IDH3a expression

**Figure 1:**
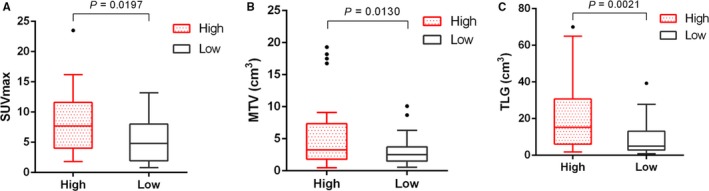
Box‐Whisker plots of SUV_max_, MTV and TLG in high and low IDH3a expression groups were shown. Comparisons of semi‐quantitative indexes between these two groups were made. Two‐tailed Student's *t* test was used for statistical analysis. (A) SUV_max_ of high IDH3a expression group is higher than that of low expression group; (B) MTV of high IDH3a expression group is higher than that of low expression group; (C) TLG of high IDH3a expression group is higher than that of low expression group. For each group, median levels of SUV_max_, MTV and TLG and interquartile ranges are illustrated by box plot, and the whiskers show minimum and maximum value representing ranges. Outliers were shown as points. SUV_max_, maximum standard uptake value; MTV, metabolic tumor volume; TLG, total lesion glycolysis

**Figure 2:**
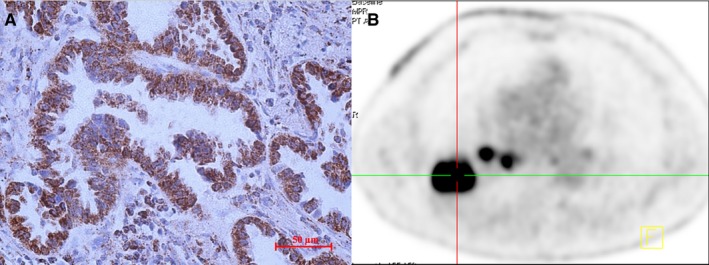
PET imaging of a case with high IDH3a expression. (A) The right inferior lung lobe of a 68‐year‐old man showed an adenocarcinoma with high IDH3a expression (immunohistochemical staining ×400; scale bar, 50 μm). (B) [^18^F]‐FDG PET/CT scan showed significant accumulation of [^18^F]‐FDG in the tumor (SUV_max_ = 15.9, MTV = 10.2 cm^3^ and TLG = 86.3 cm^3^). IDH, isocitrate dehydrogenase; SUV_max_, maximum standard uptake value; MTV, metabolic tumor volume; TLG, total lesion glycolysis

**Figure 3:**
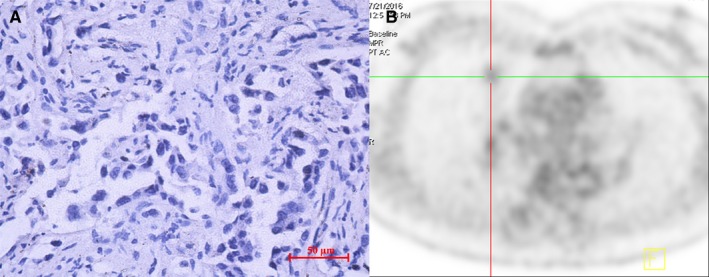
PET/CT imaging of a case with low IDH3a expression. (A) The right middle lung lobe of a 53‐year‐old woman showed an adenocarcinoma with low IDH3a expression (immunohistochemical staining ×400; scale bar, 50 μm). (B) [^18^F]‐FDG PET/CT scan showed slight accumulation of [^18^F]‐FDG in the tumor (SUV_max_ = 1.8, MTV = 3.2 cm^3^, TLG = 2.7 cm^3^). IDH, isocitrate dehydrogenase; SUV_max_, maximum standard uptake value; MTV, metabolic tumor volume; TLG, total lesion glycolysis

IDH3a immunoreactivity that was readily detected in the cytoplasm of tumor cell was considered to be a positive result. Tumors with a final immunohistochemically staining score ≥4 were considered to specific protein overexpression and included in high expression group, otherwise included in low expression group. A total of 65 patients were divided into high (31 cases) and low (34 cases) IDH3a expression groups finally. Semi‐quantitative indexes of [^18^F]‐FDG uptake including SUV_max_, MTV and TLG of the high expression group were 8.0 ± 5.0, 5.5 ± 5.3 cm^3^ and 22.1 ± 21.2 cm^3^, that of low expression group were 5.4 ± 3.8, 3.0 ± 2.1 cm^3^ and 9.3 ± 9.1 cm^3^, respectively. The difference between these two groups was significant (*P* < .05). No significant differences were found in terms of age, sex, or TNM category, but not for tumor size (Table 1 and Figure 1). Typical cases with high or low IDH3a expression and corresponding PET/CT imaging (Figures 2 and 3) are shown.

**Table 1:** Comparison of clinicopathological characteristics between low and high IDH3a expression groups of 65 patients

Characteristic	Low expression group (n = 34)	High expression group (n = 31)	*t*	*P‐value*
Age	60.6 ± 10.1	59.2 ± 9.6	−0.581	.5631
Gender			−0.835	.4071
Male	14	16		
Female	20	15		
Tumor size(mm)	20.3 ± 9.7	28.5 ± 14.1	2.781	.0071
TNM stage			−0.020	.9844
I, II	29	26		
III, IV	5	5		

Abbreviations: IDH3, isocitrate dehydrogenase; TNM, tumor‐node‐metastasis.

### 3.2 Correlation between GLUT1, HK2 expression and IDH3a expression

**Figure 4:**
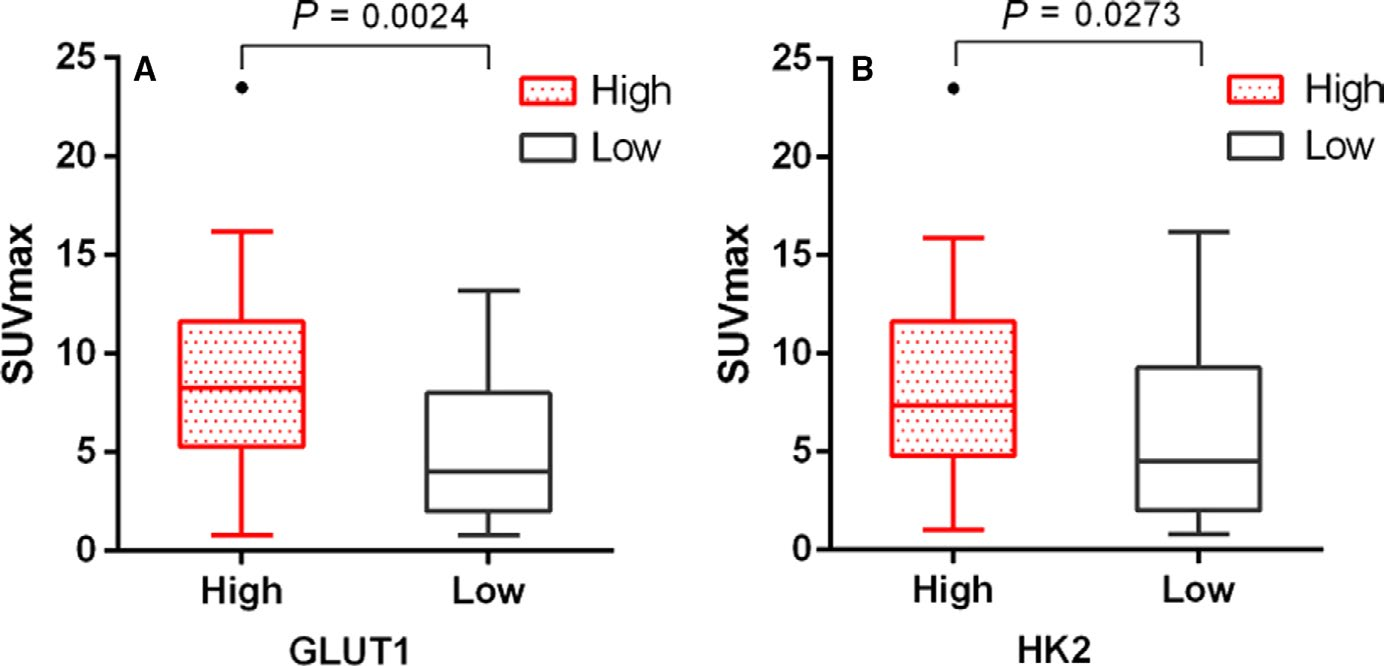
Box‐Whisker plots of SUV_max_ in high and low GLUT1 or HK2 expression groups were shown. Comparisons of SUV_max_ between these two groups were made. Two‐tailed Student's *t* test was used for statistical analysis. (A) according to GLUT1 expression scores, SUV_max_ of high expression group was significantly higher than that of low expression group; (B) according to HK2 expression scores, SUV_max_ of high expression group was significantly higher than that of low expression group. For each group, median levels of SUV_max_ and interquartile ranges are illustrated by box plot, the whiskers show minimum and maximum value representing ranges. Outliers were shown as points. SUV_max_, maximum standard uptake value; GLUT1, glucose transporter 1; HK2, hexokinase 2

**Figure 5:**
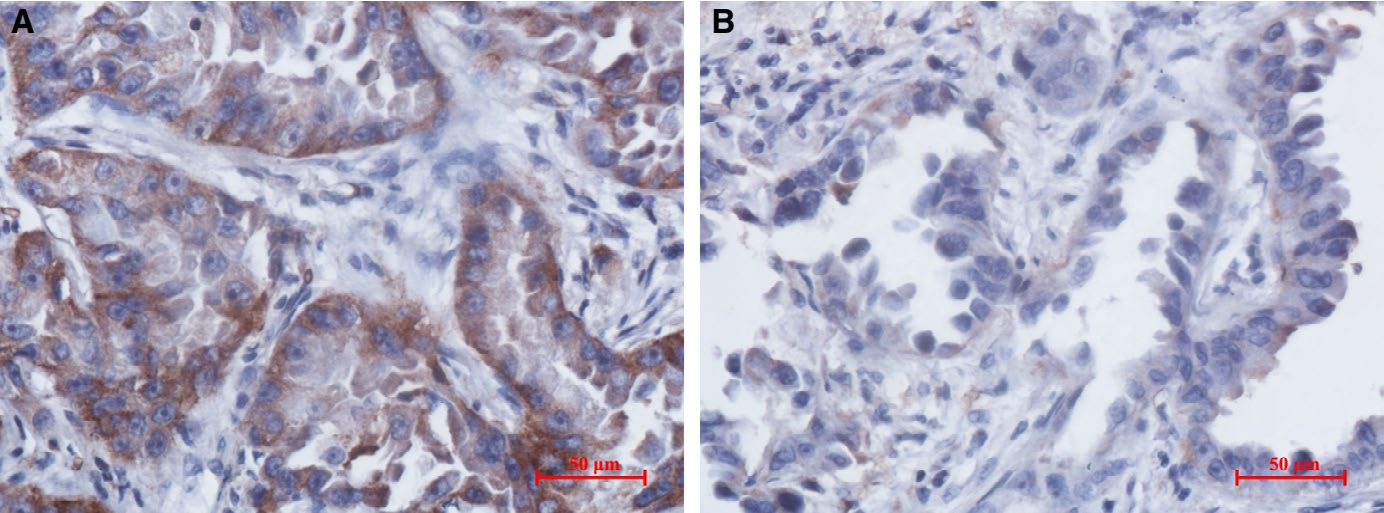
A case of IDH3a high expression group showed GLUT1 high expression but not for HK2. GLUT1 and HK2 expression of a 59‐year‐old woman in high IDH3a expression group with lung adenocarcinoma in right upper lobe showing significant accumulation of [^18^F]‐FDG (SUV_max_ = 11.8). (A) GLUT1 was highly expressed (immunohistochemical staining ×400; scale bar, 50 μm); (B) HK2 expression was low (immunohistochemical staining ×400; scale bar, 50 μm). SUV_max_, maximum standard uptake value; GLUT1, glucose transporter 1; HK2, hexokinase 2

**Figure 6:**
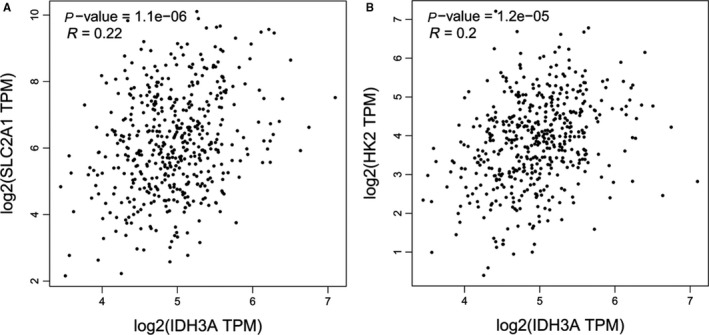
Relationship between IDH3a expression and related indicators (GLUT1 and HK2) were studied with bioinformatics analysis using GEPIA. (A) Mild correlation with coefficient of 0.22 was found between IDH3a with GLUT1. (B) Mild correlation with coefficient of 0.2 was found between IDH3a with HK2

GLUT1 and HK2 levels were investigated by immunohistochemical analysis of 65 primary tumors. According to GLUT1 expression scores, 65 patients were divided into high (26 cases) and low (39 cases) expression groups. SUV_max_ of high and low expression groups were 8.7 ± 5.0 and 5.2 ± 3.8, respectively (*P* < .05; Figure 4A). According to the HK2 expression scores, 65 patients were divided into high (30 cases) and low expression groups (35 cases). SUV_max_ of high and low expression groups were 7.9 ± 4.9, and 5.5 ± 4.0, respectively (*P* < .05; Figure 4B). A moderate correlation was confirmed between IDH3a and GLUT1 expression (*P* < .05) with Phi coefficient of 0.478, but not between IDH3a and HK2 (Table 2, Figure 5). These results indicate that IDH3a might promote [^18^F]‐FDG uptake in lung adenocarcinoma by the upregulation of GLUT1 expression. The relationship between IDH3a expression and related indicators (GlLUT1 and HK2) were also studied with bioinformatics analysis using GEPIA. Mild correlation with coefficient of 0.22 and 0.2 were found between IDH3a with GLUT1 and HK2, respectively (Figure 6).

**Table 2:** Correlation between GLUT1, HK2 expression and IDH3a expression

		IDH3a expression		*χ* ^2^	*P‐value*	Phi
		High	Low			
GLUT1 expression	High	20	6	14.84	.0001	0.478
	Low	11	28			
HK2 expression	High	15	15	0.1189	.7302	0.043
	Low	16	19			

Abbreviations: GLUT1, glucose transporter 1; HK2, hexokinase 2; IDH, isocitrate dehydrogenase.

### 3.3 IDH3a downregulation lead to reduced expression of AKT and GLUT1

**Figure 7:**
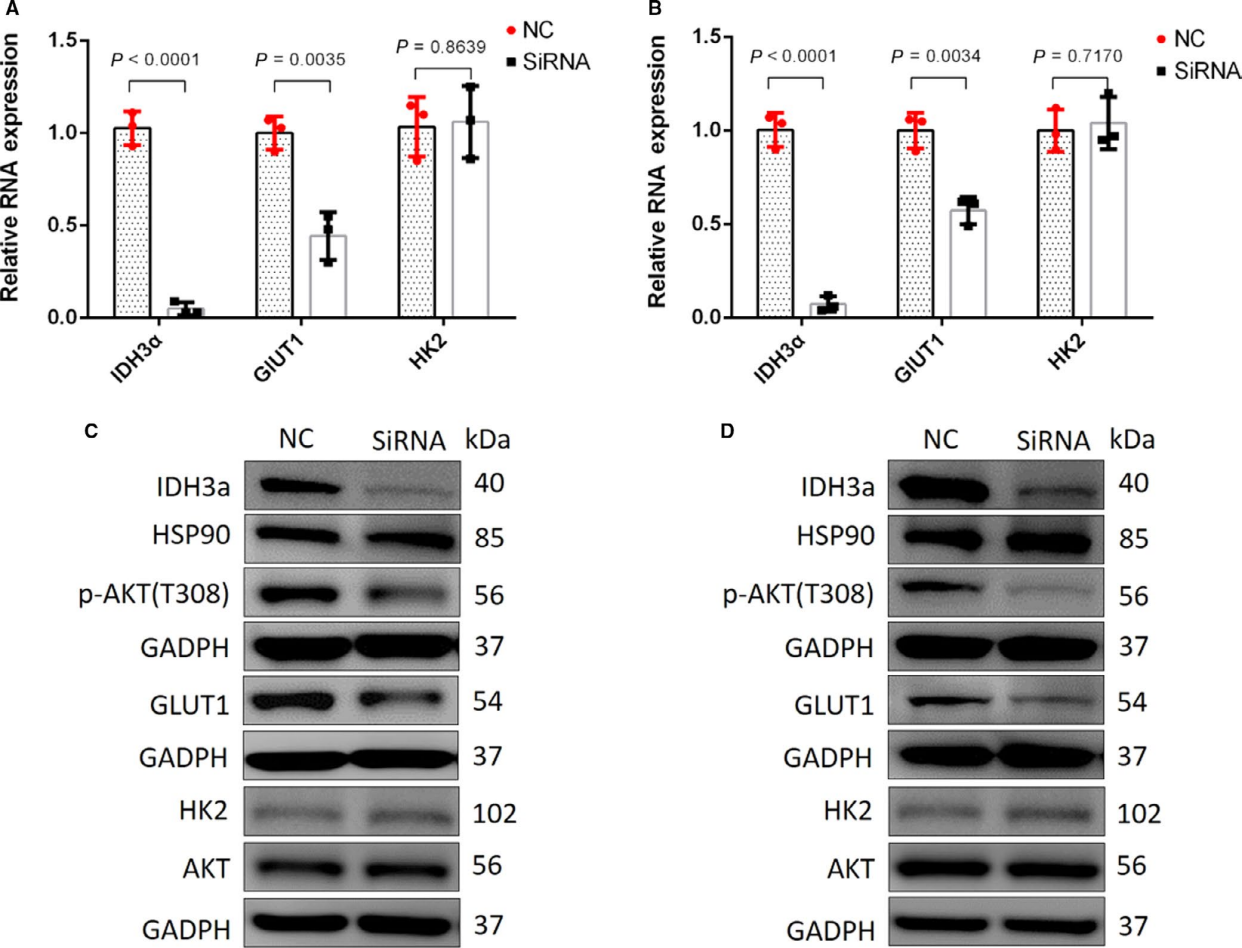
After silencing of *IDH3a, GLUT1*, and *HK2* mRNA expression were measured by RT‐PCR. GLUT1, HK2, p‐AKT, and AKT level were detected using western blotting of A549 and H1299. (A, B) The relative expression of target genes (*IDH3ɑ, GLUT1, and HK2*) of A549 and H1299 were calculated using the 2^−ΔΔCt^ method, using β‐actin as the internal reference. The fold change of treated group comparing to NC group was obtained after log‐transformation. Independent t‐test was used for statistical analysis. All tests were performed in independent triplicate experiments. The results showed a significant decrease in *GLUT1*, but not in *HK2* mRNA expression. Measurements were performed in three independent experiments; (C, D) Western blotting of A549 and H1299 showed that the expression of IDH3a was significantly decreased after silencing of IDH3a, corresponding p‐AKT and GLUT1 expression levels were downregulated, while the expression of HK2 remained unchanged. All experiments were performed in triplicate. IDH, isocitrate dehydrogenase; GLUT1, glucose transporter 1; HK2, hexokinase 2

Three siRNA sequences targeting *IDH3a* were designed to downregulate *IDH3a* expression. SiRNA S1 was selected as the chosen siRNA tool for its high transfection efficiency demonstrated by real‐time PCR and western blot. After silencing of *IDH3a*, RT‐PCR results showed a significant decrease of *GLUT1* mRNA compared to normal control group in transfected A549 or H1299 cells (*P* < .05; Figure 7A,B). There was no significant change in *HK2* mRNA. Followed western blot analysis revealed a significant decrease in IDH3a protein expression after *IDH3a* interference. Protein expression levels of p‐AKT and GLUT1 were downregulated, while HK2 expression remained unchanged (Figure 7C,D).

### 3.4 Effect of IDH3a knockdown on cellular glucose uptake, metabolites produce, and cell proliferation

**Figure 8:**
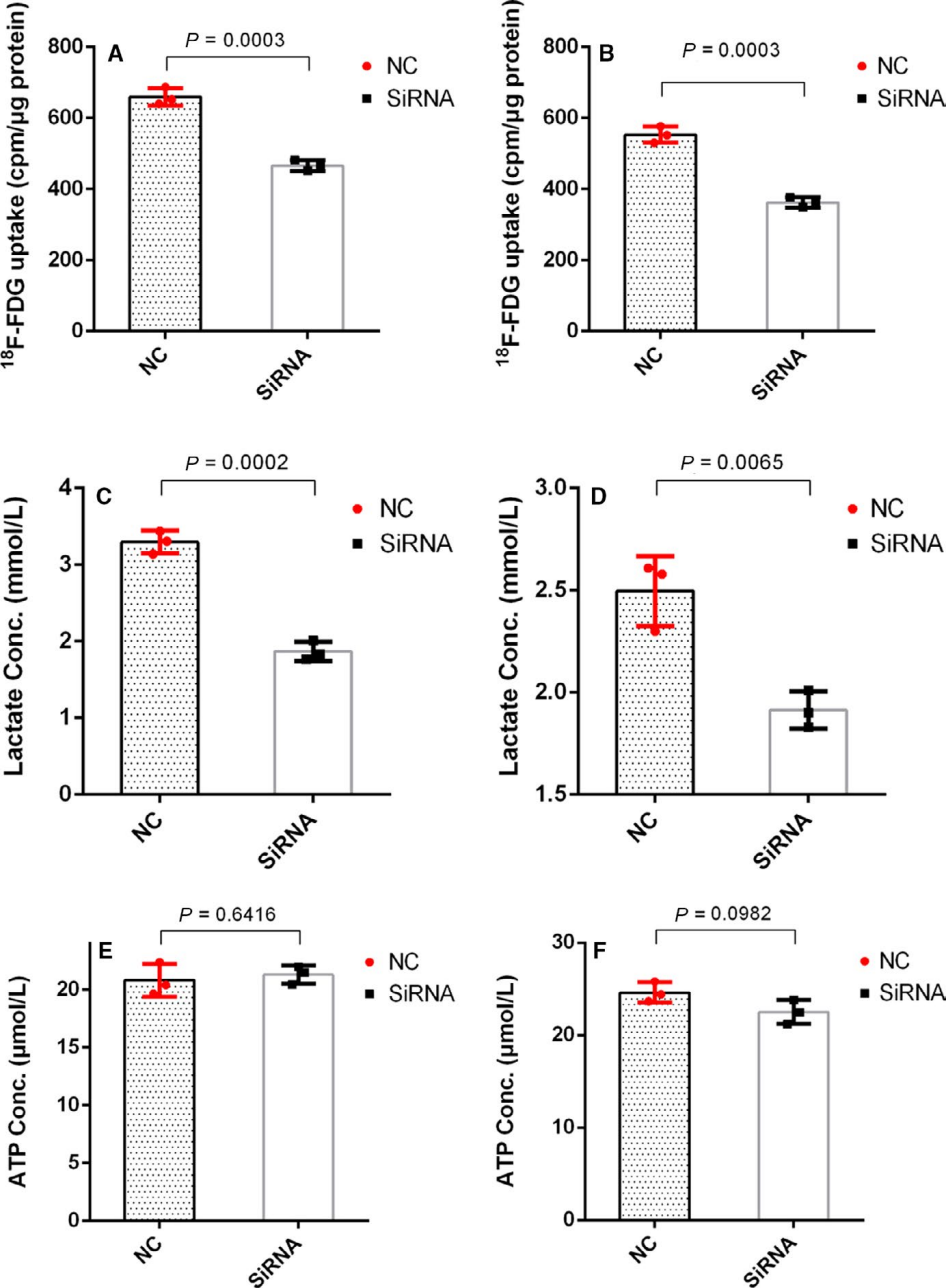
Effect of IDH3a knockdown on cellular glucose uptake and metabolites production. All experiments were performed in biological triplicate. Independent *t* test was used for statistical analysis. (A, B) Cellular [^18^F]‐FDG uptake was significantly decreased after IDH3a siRNA transfection; (C, D) lactate level in the culture medium of IDH3a siRNA transfected A549 and H1299 cells was significantly decreased; (E, F) ATP level of IDH3a siRNA transfected A549 and H1299 cells was unchanged. IDH, isocitrate dehydrogenase; ATP, adenosine triphosphate

**Figure 9:**
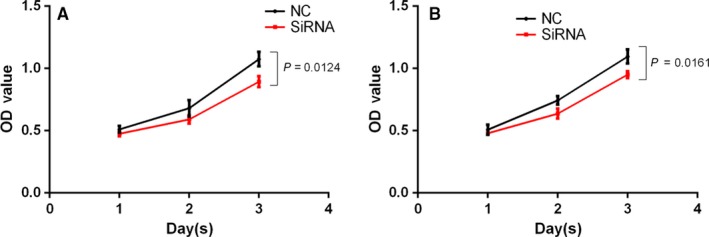
Effect of IDH3a knockdown on cell proliferation. All experiments were performed in biological triplicate. Independent *t* test was used for statistical analysis. SiRNA transfection resulted in significant growth inhibition compared with the control group (A: A549; B: H1299)

We performed a [^18^F]‐FDG uptake assays to determine if reduced IDH3a expression had an effect on glucose uptake. After *IDH3a* siRNA transfection in A549 or H1299 cells, cellular [^18^F]‐FDG uptake was significantly decreased (Figure 8A,B). Cellular ATP and lactate levels in the medium were measured to reflect changes in cellular metabolism. After *IDH3a* siRNA transfection, Lactate levels in the medium decreased significantly (Figure 8C,D); however, ATP levels remained unchanged (Figure 8E,F). To assess the potential relevance of IDH3a as a therapeutic target, in vitro knockdown experiments were performed. Significant growth inhibition was observed in siRNA‐treated lung cancer cells (Figure 9).

## 4 DISCUSSION

Lung cancer is one of the most common cancers worldwide, its morbidity and mortality are increasing year by year.^12^, ^13^, ^14^ Lung adenocarcinoma is the most common pathological type of lung cancer.^15^ Rapid progression, easy to metastasize and chemoresistance are the challenges faced by treatment.^16^, ^17^ The discovery and application of new molecular targets that reflect tumor biological behavior, and even potentially be a therapeutic target, may be helpful.

As an important enzyme in the Krebs cycle, IDH in humans is encoded by five genes, forming three subtypes, including IDH1, IDH2, and IDH3. IDH1 plays a role in cytoplasm and peroxisomes, while IDH2 and IDH3 are present in mitochondria. IDH1 and IDH2 gene mutations have been detected in gliomas, acute myeloid leukemia etc, resulting in inactivation of IDH leading to tumorigenesis.^18^, ^19^ IDH3 has been demonstrated to play an important role in tumor metabolism reprogramming in recent studies,^3^, ^4^, ^6^ and also considered as lung cancer gene.^20^ [^18^F]‐FDG PET/CT is a less invasive imaging tool widely used in the diagnosis and staging of malignant tumors. The distribution of [^18^F]‐FDG detected by PET/CT reflects glucose uptake in tumors. Studies have found that most malignant tumors increase the efficiency of glucose uptake by upregulating the expression of GLUT. GLUT1 is highly expressed in non‐small cell lung cancer and is associated with [^18^F]‐FDG uptake.^21^, ^22^ Upon entry, glucose is converted to 6‐glucose phosphate under HK, which is the first stage of glycolysis. HK plays a dominant role in glycolysis by promoting the coupling of ATP with glucose phosphorylation. Wang et al showed that silencing HK2 expression and inhibiting the expression of 2‐DG inhibited tumor cell proliferation and tumor growth.^23^ As a core factor of PI3K‐AKT‐mTOR signaling pathway, AKT plays a prominent role in increasing glucose uptake of tumors. GSK2141795, as an AKT inhibitor, has significant antitumor effect.^24^, ^25^, ^26^, ^27^, ^28^

In this study, the correlation between IDH3a expression and the semi‐quantitative indexes of [^18^F]‐FDG PET/CT imaging including SUV_max_, MTV and TLG, were analyzed. SUV_max_ reflects the most active part of the tumor, MTV indicates the volume of metabolically active tumor, and TLG reflects both metabolic activity and volume. All of these were used to reflect the extent of tumor [^18^F]‐FDG uptake in different aspects. We found that patients with high IDH3ɑ expression are associated with higher uptake of [^18^F]‐FDG, which means that IDH3a plays an important role in aerobic glycolysis of malignant tumors. This result is consistent with the findings of Zeng et al,^3^ and was related to the study by Zhou et al, 29 who found that abnormal expression of lactic dehydrogenase is correlated with the enhancement of aerobic glycolysis. We also found the size of tumor was associated with IDH3a expression, suggesting that IDH3ɑ may affect tumor proliferation. The expression of IDH3ɑ also correlated with the expression of GLUT1, which means that IDH3ɑ may promote glycolysis of lung adenocarcinoma by upregulating GLUT1. The bioinformatics analysis results, other than verified the correlation between IDH3a with GLUT1, also found the mild correlation between IDH3a with HK2, which was not consistent with immunohistochemistry study results. Previous studies also found the RNA expression did not always correlate with protein expression.^30^, ^31^, ^32^

Our study further explored the cellular mechanisms involved in IDH3a affecting glucose uptake. We identified key factors affecting glucose uptake in lung cancer cell, including GLUT1, HK2, and p‐AKT. RT‐PCR and western blot results confirmed that downregulation of IDH3ɑ expression resulted in the downregulation of p‐AKT and GLUT1, but HK2 remained unchanged. This indicates that IDH3a affects glucose uptake through an AKT/GLUT1 pathway. These results are consistent with the studies in which resveratrol inhibits ovarian cancer cells and apigenin inhibits hepatoma cells.^33^, ^34^ After silencing DH3ɑ expression, [^18^F]‐FDG uptake of tumor cells reduced, confirming our previous in vivo study. The downregulation of IDH3a affects the production of metabolite, we found lactate in the culture medium was reduced to a certain extent, while the ATP levels did not change significantly, which may be related to the compensatory mechanisms of tumor energy production.

The driver mutation causing aberrant expression of IDH3a in different malignant tumor has not been clarified. IDH3a was considered to be a target gene of NF‐kB^35^ and IDH3α‐cSHMT signaling axis represents a novel mechanism of metabolic adaptation in glioblastoma.^5^ Targeting tumor glycolytic provides new ideas for cancer treatment.^36^, ^37^ Tributyltin is an organotin compound that is an endocrine disruptor similar to androgen. The tumor suppressive effect of tributyltin was focused on in recent studies.^38^, ^39^ Yamada et al found in a series of studies that low levels of tributyltin specifically bind to IDH3ɑ to inhibit enzyme activity,^40^, ^41^ which may lead to new strategies on anti‐tumor metabolic therapy targeting IDH3ɑ. The relatively small and available sample of this study is a limitation. If more patients were included, a linear correlation analysis can be performed between IDH3a and GlUT1 expression. This study will be more convincing if qPCR and Western blot analysis are performed on frozen tumor tissue from patients. Another limitation is the lack of animal tumor model study. The conclusion may be more convincing if it can be demonstrated that IDH3a interference or inhibitor can affect tumor glucose uptake in animal tumor model with [^18^F]‐FDG PET scanning.

## 5 CONCLUSIONS

Lung adenocarcinoma patients with high expression of IDH3α showed more significant glucose uptake. IDH3α affect glucose uptake by targeting GLUT1 through AKT pathway. Downregulation of IDH3α can decrease glucose uptake and lactate production in lung adenocarcinoma tumor cells.

## CONFLICT OF INTEREST

The author reports no conflicts of interest in this work.
