# Supplementary material for: Effect of IDH3a on glucose uptake in lung adenocarcinoma: A pilot study based on [18F]FDG
Source: Cancer Med. 2019 Jul 29;8(11):5341–51. doi: 10.1002/cam4.2421 (PMC6718547; doi:10.1002/cam4.2421)
Supplement: Supplementary file 1 [file CAM4-8-5341-s001.docx]

**Effect of IDH3a on glucose uptake in lung adenocarcinoma: a pilot study based on [^18^F]FDG**


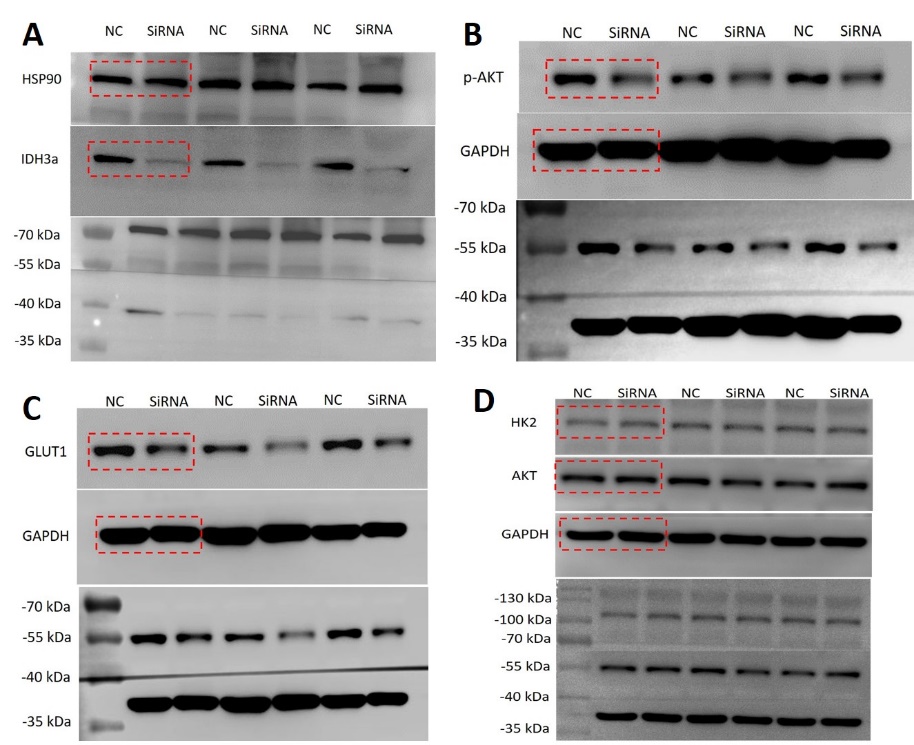


**Figure S1:** Western blotting (n=3) were performed in A549 cell line. (A) Western blotting of IDH3a, HSP90 was used as loading control protein^42,43^. (B) Western blotting of p-AKT, GAPDH was used as loading control protein. (C) Western blotting of GLUT1, GAPDH was used as loading control protein. (D) Western blotting of HK2 and AKT, GAPDH was used as loading control protein. Red dotted boxes indicated the site for cropping. The lower part of each picture was an overlay white light (for imaging of marker ladder) and chemiluminescence images.


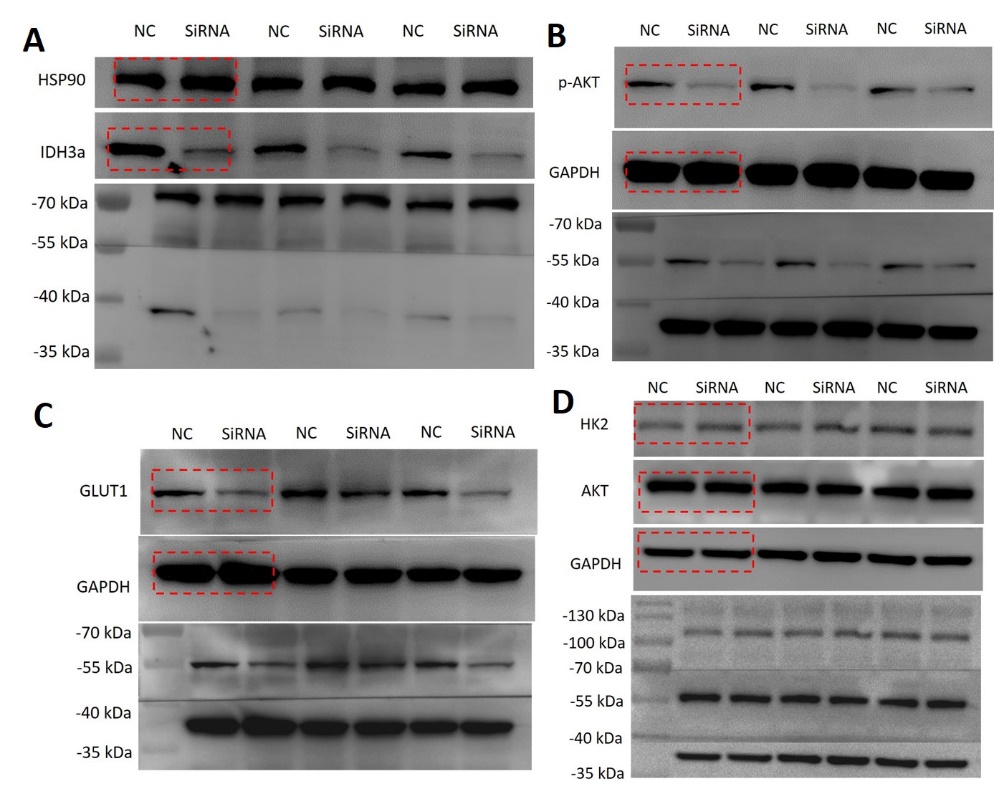


**Figure S2:** Western blotting (n=3) were performed in H1299 cell line. (A) Western blotting of IDH3a, HSP90 was used as loading control protein. (B) Western blotting of p-AKT, GAPDH was used as loading control protein. (C) Western blotting of GLUT1, GAPDH was used as loading control protein. (D) Western blotting of HK2 and AKT, GAPDH was used as loading control protein. Red dotted boxes indicated the site for cropping. The lower part of each picture was an overlay white light (for imaging of marker ladder) and chemiluminescence images.

**Reference**

1. Shimamura T, Chen Z, Soucheray M, et al. Efficacy of BET Bromodomain Inhibition in Kras-Mutant Non-Small Cell Lung Cancer. *Clin Cancer Res.* 2013;19(22):6183-6192.
2. de Thonel A, Vandekerckhove J, Lanneau D, et al. HSP27 controls GATA-1 protein level during erythroid cell differentiation. *Blood.* 2010;116(1):85-96.
